# NKT cells contribute to basal IL-4 production but are not required to induce experimental asthma

**DOI:** 10.1371/journal.pone.0188221

**Published:** 2017-11-28

**Authors:** Christopher G. McKnight, Suzanne C. Morris, Charles Perkins, Zhenqi Zhu, David A. Hildeman, Albert Bendelac, Fred D. Finkelman

**Affiliations:** 1 Division of Immunology, Allergy and Rheumatology, Department of Medicine, College of Medicine, University of Cincinnati, Cincinnati, Ohio, United States of America; 2 Department of Medicine, Cincinnati Veterans Affairs Medical Center, Cincinnati, Ohio, United States of America; 3 Division of Immunobiology, Cincinnati Children’s Hospital and Medical Center, Cincinnati, Ohio, United States of America; 4 Committee on Immunology, The University of Chicago, Chicago, Illinois, United States of America; 5 Department of Pathology, The University of Chicago, Chicago, Illinois, United States of America; Centre National de la Recherche Scientifique, FRANCE

## Abstract

CD1d-deficiency results in a selective deletion of NKT cells in mice that is reported to prevent murine allergic airway disease (AAD). Because we find 2–3 fold lower basal IL-4 production in CD1d^-^ mice than in wild-type (WT) mice, we hypothesized that the contribution made by NKT cells to AAD would depend on the strength of the stimulus used to induce the disease. Consequently, we compared CD1d-deficient mice to WT mice in the development of AAD, using several models of disease induction that differed in the type and dose of allergen, the site of sensitization and the duration of immunization. Surprisingly we found equivalent allergic inflammation and airway disease in WT and CD1d^-^ mice in all models investigated. Consistent with this, NKT cells constituted only ~2% of CD4^+^ T cells in the lungs of mice with AAD, and IL-4-transcribing NKT cells did not expand with disease induction. Concerned that the congenital absence of NKT cells might have caused a compensatory shift within the immune response, we administered an anti-CD1d monoclonal Ab (mAb) to block NKT function before airway treatments, before or after systemic sensitization to antigen. Such Ab treatment did not affect disease severity. We suggest that the differences reported in the literature regarding the significance of NKT cells in the induction of allergic airway disease may have less to do with the methods used to study the disease and more to do with the animals themselves and/or the facilities used to house them.

## Introduction

Type 2 cytokines, particularly IL-4 and IL-13, are important in the pathogenesis of human asthma and murine allergic airway disease, which is a useful, albeit imperfect, model of human asthma [1]. Human asthma is associated with increased basal IL-4 and IL-13 levels in the airway [2–5], and segmental airway challenge with allergen induced IL-4 and IL-13 responses [6–8]. Additionally segmental airway challenge with IL-4 has been reported to induce airway eosinophilia and airway hyperresponsiveness (AHR) within 24 hours [9]; gain-of-function allelic variants of the IL-4 and IL-4Rα genes impart an increased risk of asthma to carriers [10, 11]; and blocking IL-4Rα through administration of anti-IL-4Rα mAb has shown good efficacy in improving pulmonary function and decreasing exacerbations of human asthma in phase 2b trials [12]. Analogously, mice deficient in IL-4 fail to develop AAD in response to most allergens and immunization protocols [13, 14]; airway inoculation with IL-4 or IL-13 (or genetic overexpression of either cytokine) can induce airway eosinophilia, goblet cell metaplasia and AHR [15–17]; and treatment with antagonists of IL-13 or IL-4Rα can usually suppress established AHR [18–21].

In contrast to the general consensus about the importance of type 2 cytokines in human asthma and murine AAD, there is lack of agreement about how allergens induce the pulmonary Th2 response. Although nearly all reports indicate a requirement for conventional CD4^+^ T cells [22], the cellular and molecular pathways that lead these cells to differentiate into Th2 cells are uncertain. Because IL-4 promotes Th2 differentiation in naïve T cells [23, 24], because NKT cells can rapidly secrete large quantities of IL-4 [25] and because thymic selection of NKT cells depends on CD1d (an atypical MHC class I molecule that associates with β_2_-microglobin for normal function) [26], β_2_-microglobin-deficient mice were used several years ago to investigate the significance of NKT cells in generating AAD and the associated Th2 response. Murine AAD developed to approximately the same extent in allergen-immunized WT and β_2_-microglobin-deficient mice, which lack most NKT cells [27, 28].

However, more recent studies that used mice deficient in CD1d or Vα14 (the TCRα chain expressed by murine iNKT cells) reported the opposite result: deficient mice failed to develop AAD whether immunized with ovalbumin (OVA) or more potent allergens [29–31]. Failure to develop AAD was reported to result from an inadequate IL-13 response, and IL-13 inoculation was found to rescue pulmonary allergic inflammation and AHR in CD1d-deficient mice [29]. Additionally, other publications have demonstrated that inhalation of a purified NKT cell ligand can induce NKT cell-dependent AHR in the absence of conventional CD4^+^ T cells [32] and that combining synthetic NKT ligands with protein antigens can contribute to the activation of conventional CD4^+^ T cells in AAD [33, 34]. Notably the disparity in results between β_2_-microglobin-deficient mice and those mice lacking either CD1d or Vα14 may have been partially explained by a report that found that CD1d can be expressed to some extent in the absence of β_2_-microglobin and that AAD was induced in β_2_-microglobin-deficient mice but not in CD1d-deficient mice [35]. Not all investigators, however, have reported that CD1d is required for the development of murine AAD; two groups observed relatively normal allergen-induced airway eosinophilia and/or airway pathology in CD1d-deficient mice, although neither group evaluated whether these mice developed AHR [36, 37].

Controversy about the role of NKT cells in AAD has also extended to studies of human asthma. A report by one group that ~60% of pulmonary CD3^+^CD4^+^ cells in patients with moderate to severe asthma were NKT cells, rather than classical MHC II-restricted T cells [38], was criticized by other investigators on a technical basis [39]. And, a second group reported that only ~2% of CD4^+^ T cells in the lung of both normal individuals and asthmatics were NKT cells [40]. In response, the first group published evidence that NKT cells comprised a higher percentage of all CD4^+^ T cells in the lungs of severe asthmatics compared to non-severe asthmatics. Notably all but one of the severe asthmatics in this study demonstrated <5% NKT cells [41]. Perhaps the most difficult issue in this controversy for those who favor the importance of NKT cells in the pathogenesis of asthma and experimental AAD has been the mechanism by which immunization with a protein antigen, such as OVA, activates these cells which preferentially recognize lipid antigens [26].

Because of this specificity and the paucity of NKT cells in AAD, we initiated our study by hypothesizing that NKT cells might be an important basal source of IL-4 and that such IL-4 production could be important in promoting AAD when the airway encounters allergen at a limiting dose for a limited amount of time. Indeed, we found a 2–3 fold lower basal level of IL-4 production in CD1d-deficient mice. Consequently, we compared CD1d-deficient mice to WT mice in several different models of disease induction, believing that the significance of the IL-4 produced by NKT cells would be specific to the experimental conditions used for allergic sensitization and/or induction of lung disease. Surprisingly we did not identify any model of AAD in which the presence of CD1d, or NKT cells, facilitated the induction of disease. As a result, we suggest that the differences reported in the literature regarding the significance of NKT cells in the induction of allergic airway disease may have less to do with the methods used to study the disease and more to do with the animals themselves and/or the facilities used to house them.

## Materials and methods

### Mice and immunological reagents

BALB/c CD1d^-/-^ breeding pairs were purchased from The Jackson Laboratory (Bar Harbor, ME) and matched for age and sex with in-house bred BALB/c wildtype mice for all experiments. All mice were bred in the AAALAC-approved barrier-isolation specific pathogen-free animal facility at Cincinnati Children’s Hospital Medical Center, accessed food and water ad libitum and monitored daily by the veterinary staff for health and well-being. We performed all experimentation while conscientiously minimizing animal stress and suffering. Mice were sacrificed through injection of intraperitoneal pentobarbital and xylazine. All experiments were approved by the Cincinnati Children’s Hospital Medical Center Institutional Animal Care and Use Committee. Mice were immunized with house dust mite extract (HDM) (*D*. *pteronyssinus*, Greer Labs, Lenoir, NC), grade V OVA (Sigma, St. Louis, MO), or α-galactosylceramide (α-GalCer) (KRN700, Enzo, Farmingdale, NY). Clone 20H2 (also known as HB-323), a hybridoma that secretes rat IgG1 anti-CD1d mAb (Roark et al., 1998) was purchased from the American Type Culture Collection (Manassas, VA). GL113 (rat IgG1 anti-E. coli α-galactosidase) was used as an isotype control. 20H2, GL113 and the complementary pair of anti-IL-4 clones (BVD41D11 and BVD624G2.3) were grown in the ascites of Pristane-primed athymic nude mice and the mAbs were purified by ammonium sulfate fractionation followed by DE-52 ion exchange chromatography.

### Measurement of IL-4 and IFN-γ production in vivo

This was achieved using the *In Vivo* Cytokine Capture Assay (IVCCA) as previously described [42]. Briefly, anti-cytokine monoclonal IgG antibody is injected; the clone must bind the cytokine with high affinity and at a position that prevents the cytokine from binding its receptor through steric interference. A blood sample is then taken several hours later to quantitate by ELISA the amount of circulating cytokine-IgG complexes.

### Intratracheal inoculation

Mice were anesthetized by i.p. injection of ketamine plus xylazine and allowed to hang from a string by their upper teeth on a 60 degree incline. Their tongues were gently withdrawn with a blunt forceps and held to prevent ingestion. 40 μl of solution was pipetted onto the base of the tongue, and the nose was occluded to encourage inhalation. Mice recovered in a prone position under supplemental oxygen.

### Intranasal inoculation

Mice were anesthetized with isoflurane, then held by the skin of the posterior neck. Solution was slowly pipetted into the nares as spontaneous breaths allowed, and mice were observed vertically until inhalation of solution was complete.

### HDM challenge protocol

Mice were inoculated i.t. (intratracheally) with saline or the specified quantity of total protein of HDM extract in a volume of 40 μl saline every other day, either 6 or 9 times.

### High dose OVA-based protocol

100 μg of OVA adsorbed to 4 mg of alum was injected i.p. on day 0 and 7. 100 μg of OVA in 50 μl saline was then inoculated i.t. daily on day 15–24.

### Lower dose OVA-based protocol

50 μg of OVA adsorbed to 4 mg of alum was injected i.p. on day 0. 50 μg of OVA in 50 μl saline was then delivered i.n. (intranasally) daily on day 7, 8, and 9.

### Ovalbumin aerosol challenge protocol

BALB/c female WT and CD1d^-^ mice were immunized i.p. with 20 μg of OVA adsorbed to 4.25 mg of alum (Thermo-Pierce, Rockford, IL) on days 0 and 14. On days 28, 29 and 30, up to 8 mice were placed in a chamber and exposed for 20 min to an ovalbumin aerosol produced by nebulizing 25 ml of 1% OVA in PBS at the maximal rate with a DeVilbiss Ultra-neb 99 system. Mice were evaluated for AHR and pulmonary eosinophilia on day 31.

### NKT cell activation protocol

The NKT cell ligand, α-GalCer, (KRN7000) was dissolved at a concentration of 1 mg/ml of DMSO; then diluted in normal saline to a concentration of 40 μg of KRN7000/ml. Mice were challenged i.t. on day 0 with 2 μg of KRN7000 in 50 μl saline.

### Measurement of airway responsiveness to β–methacholine by barometric plethysmography

This was performed as described [43], except an Aeroneb Lab Nebulizer (Aerogen, Galway, Ireland) generated aerosol particles, 2.5–4 μm in diameter. Briefly, after careful system calibration, mice were placed in plethysmographic chambers and their baseline “enhanced pause” (Penh) calculated by Buxco Research System’s software (Wilmington, NC). Mice were then exposed to increasing concentrations of methacholine (Sigma, St. Louis, MO) through a series of aerosols while the system measured the Penh of each breath’s wave form and provided the average values following each dose of methacholine.

### Invasive measurement of airway responsiveness to β-methacholine by forced oscillation

This was performed as previously described [43]. Briefly, mice were: anesthetized with pentobarbital and xylazine, provided a tracheostomy through which a cannula was secured, ventilated mechanically with the flexiVent system (SCIREQ, Montreal, Canada) and paralyzed with pancuronium (Sigma, St. Louis, MO). After 2 minutes of default ventilation, 2 total lung capacity maneuvers were delivered for airway recruitment and before every exposure to aerosol. PBS was nebulized (Aeroneb Lab Nebulizer (Aerogen, Galway, Ireland)), followed by measurement of airway resistance through a series of 4 Snap-Shot perturbations, using the single compartment model of airway mechanics. A series of increasingly concentrated methacholine solutions were then administered by nebulization; airway resistance was measured 10 times after each methacholine dose and the highest value, with a coefficient of determination of at least 0.9, was used for analysis.

### Bronchoalveolar lavage (BAL)

Immediately after invasive measurement of pulmonary function, the ends of polyethylene catheters (OD = 0.97 mm Becton Dickinson, Sparks, MD) were secured into the tracheas. Lungs were lavaged 3x with 2.5 ml of Ca^2+^-free and Mg^2+^-free Hanks’ balanced salt solution (HBSS), (Lonza, Rockville, MD), that contained 0.02% EDTA and EGTA. The BAL fluid was centrifuged at 1,000 rpm for 5 minutes at 4°C and the cell pellets were resuspended in 0.5 ml cold HBSS with 10% newborn bovine serum and 0.2% NaN_3_. Cells were counted with a Coulter counter (Beckman Coulter, Brea, CA) and 2 x 10^5^ cells were centrifuged onto a glass microscopic slide using a cytocentrifuge (Thermo Scientific, Rockford, IL). Slides were stained with Giemsa stain (Sigma, St. Louis, MO), and at least 200 hematopoietic cells were counted examined microscopically. Cells were characterized as neutrophils, lymphocytes, macrophages, or eosinophils.

### Detection of CD1d on cells by flow cytometry

To evaluate CD1d expression, lymphocytes isolated from spleens of wildtype and CD1d^-/-^ mice were blocked for nonspecific staining with the anti-FcγRII/RIII receptor mAb, 2.4G2 [44]. Cells were then stained for 30 minutes at 4°C with PE-labeled anti-mouse CD1d or isotype control mAbs and with FITC-labeled anti-mouse B220 (RA3-6B2) (BD Pharmingen, San Diego, CA). Analysis was performed on a BD FacsCalibur (Becton Dickinson), and expression of CD1d on B220^+^ cells was determined with CellQuest software.

### Detection of NKT cells by flow cytometry

Wildtype and CD1d^-/-^ mice were inoculated i.t. with 50 μm of HDM extract 9 times or saline, or inoculated using the high dose OVA-based protocol above. Lungs and liver were collected 1 day after the final allergen inoculation. A lung digest was performed by incubating minced lung with 2.5 mg Liberase TL (Roche, Indianapolis, IN) and 550 μg DNAse (Sigma, St. Louis, MO) in HBSS for 30 minutes at 37°C. The digested tissue was pushed through a 100 μm mesh strainer with the plunger end of a 3 ml syringe. Cells were spun at 1,000 rpm for 5 minutes and red blood cells were then lysed with a solution of ammonium chloride and potassium bicarbonate. Cells were then washed, spun again at 1,000 rpm for 5 minutes, resuspended and counted with a Coulter counter. Nonspecific Fc receptor staining was blocked by incubating cells on ice with anti-Fc receptor mAb, 2.4G2 for 10 minutes. Cells were then stained with PE-labeled, α-GalCer-loaded CD1d-tetramers or with PE-labeled unloaded CD1d-tetramers (NIH Tetramer Core Facility, Atlanta, GA) at room temperature for 25 minutes. Cells were washed following this incubation and further stained on ice with FITC-anti-CD4, PerCP-anti-CD8, and APC-anti-TCRβ.

### Statistics

One-way ANOVA was used to assess significance when multiple groups were compared. Mann-Whitney *U* tests were used when only 2 groups were compared. Two-way ANOVA was used to assess the significance of continuous data obtained by measuring airway responsiveness. p values of <0.05 were considered statistically significant. All figures show mean ± SEM.

## Results

### Authentication of CD1d^-^ mice

**Fig 1:**
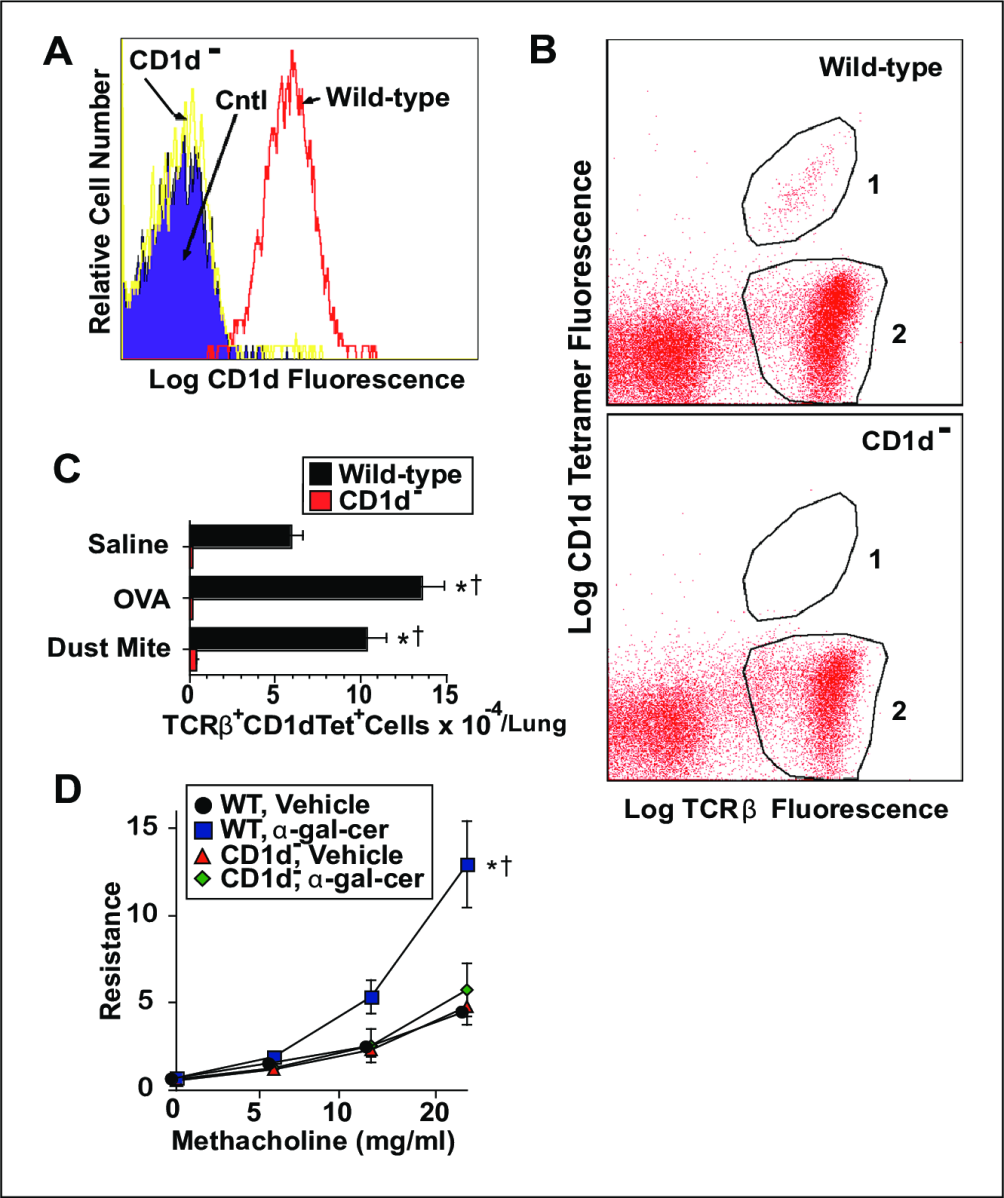
Authentication of CD1d^-^ mice. Panel A, spleen cells from WT and CD1d^-^ BALB/c mice were stained with fluorochrome-labeled anti-B220 and anti-CD1d mAbs, and analyzed for CD1d expression by B220^+^ cells (B cells) by flow cytometry. Panel B, Lymphoid cells from the lungs of WT and CD1d^-^ mice immunized with OVA were stained with fluorochrome-labeled α-GalCer-CD1d-tetramer and anti-TCRβ mAb, then analyzed by flow cytometry for CD1d-tetramer binding to TCRβ^+^ cells. In this representative sample, NKT cells lie within Gate 1, and conventional T cells lie within Gate 2. Panel C, average numbers of TCRβ^+^ α-GalCer-CD1d-tetramer^+^ cells per pair of lungs from WT and CD1d^-^ mice inoculated with saline, OVA or HDM. Panel D, WT and CD1d^-^ mice were inoculated once i.t with vehicle or α-GalCer and analyzed 2 days later for airway responsiveness. 2 experiments pooled, 6 mice/group, for Panels A-C, 9-12/group for D. * = p <0.05 as compared to saline-treated mice. † = p <0.05 as compared to CD1d^-^ mice.

Initial studies were performed to assure that our CD1d^-^ mice were actually CD1d-deficient and had the expected deficit in NKT cells. Splenocytes from WT and CD1d^-^ mice were stained with anti-B220 and anti-CD1d mAbs, then analyzed for B cell expression of CD1d by flow cytometry. B cells from WT mice showed obvious expression of CD1d while B cells from CD1d^-^ mice showed no expression of this molecule (Fig 1A). Furthermore, NKT cells isolated from the lungs of WT mice were easily detected by staining with anti-TCRβ mAb and CD1d tetramer loaded with α-GalCer. This population of cells increased modestly in WT mice upon induction of AAD, but no such cells could be reliably identified in CD1d^-^ mice (Fig 1B and 1C). Importantly WT mice, but not CD1d^-^ mice, developed AHR following i.t. inoculation with the NKT cell ligand, α-GalCer (Fig 1D). Thus, any development of AAD in our allergen-immunized CD1d^-^ mice is not likely to result from partial expression of CD1d or from a novel population of NKT cells.

### Basal IL-4 levels are reduced in CD1d^-^ mice

**Fig 2:**
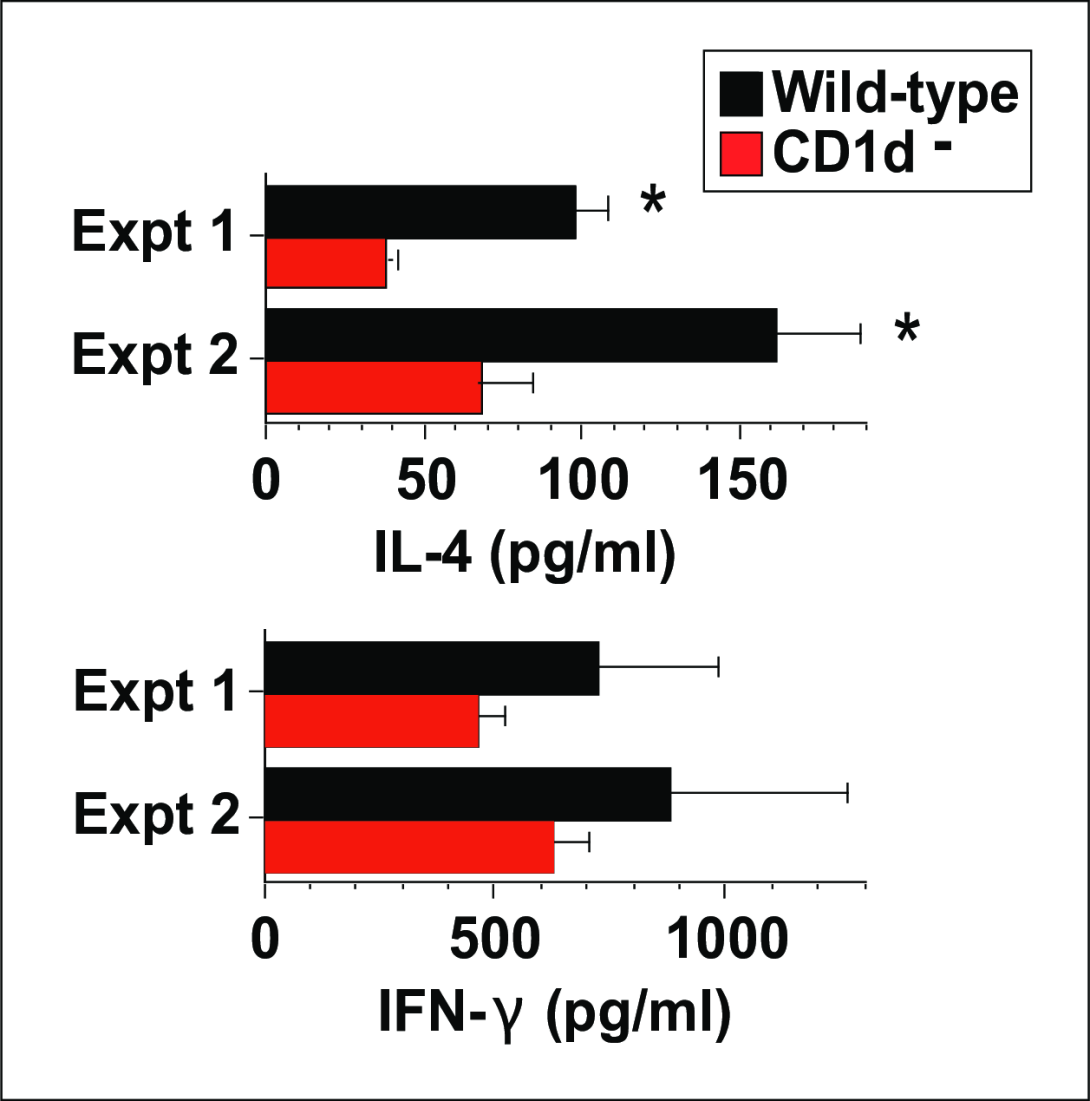
Basal IL-4 secretion is reduced in CD1d^-^ mice. IL-4 and IFN-γ secretion were measured in uninoculated mice by IVCCA. 3–4 mice/group in each experiment. * = p <0.05 as compared to CD1d^-^ mice. Similar results were observed in repeat experiments.

Because NKT cells constitutively transcribe IL-4 mRNA, we hypothesized that basal *in vivo* IL-4 secretion would be decreased in CD1d^-^ mice. We tested this hypothesis by using the highly sensitive *in vivo* cytokine capture assay (IVCCA) to measure IL-4 and IFN-γ secretion in unimmunized age- and sex-match WT and CD1d^-^ mice. The results of two separate experiments showed a significant, 2–3 fold decrease in IL-4 secretion in the CD1d^-^ mice, without a significant decrease in their secretion of IFN-γ (Fig 2).

### CD1d does not contribute to the induction of AAD in OVA-sensitized mice challenged with OVA

**Fig 3:**
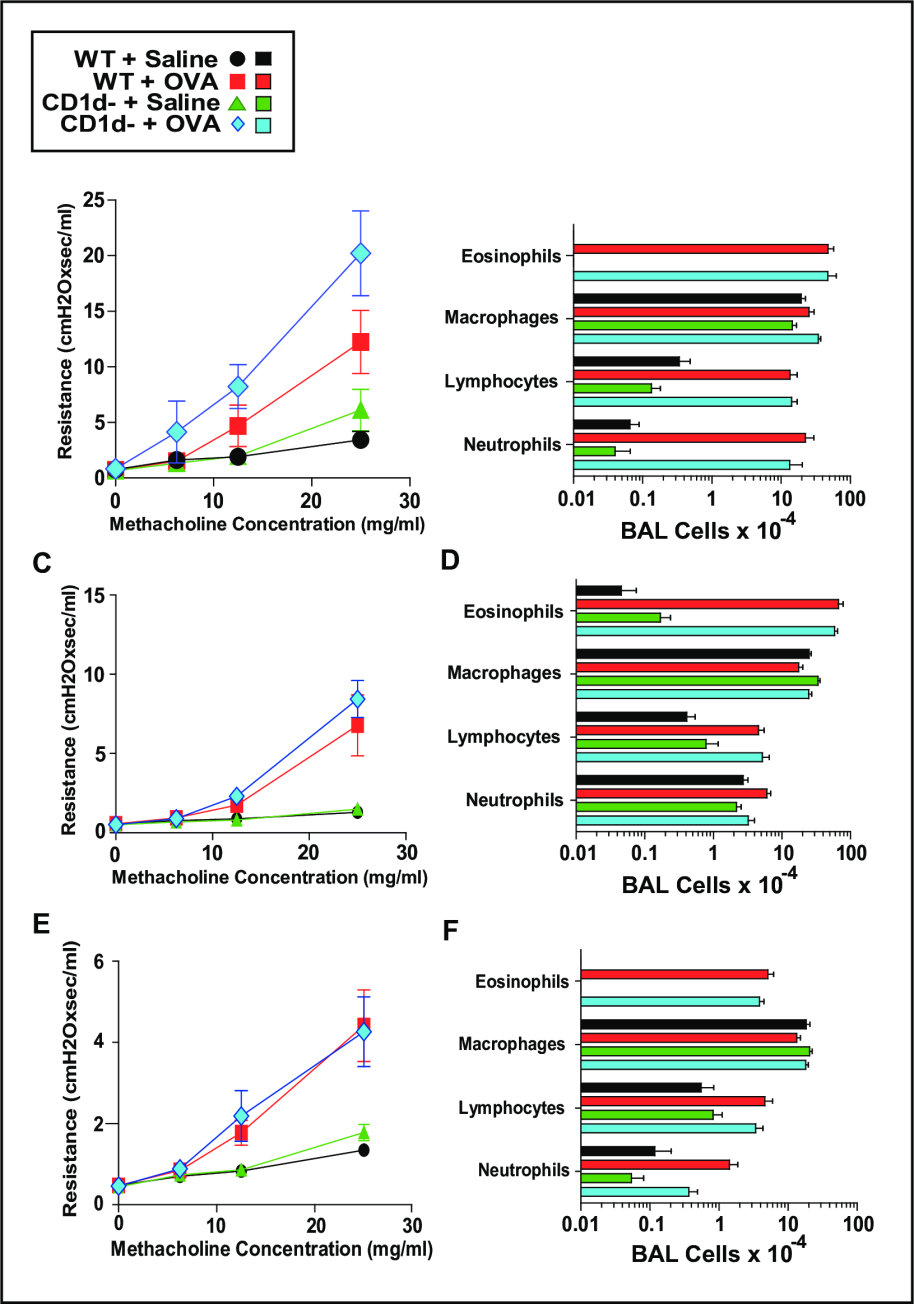
CD1d does not contribute to the induction of AAD in OVA-sensitized mice challenged with OVA. Using 3 different approaches, BALB/c WT and CD1d^-^ mice were inoculated i.p. with OVA/alum and then inhaled OVA. Panels A and B, i.p. injections were: PBS or 100 μg OVA + 4 mg alum on days 0 and 7, then airway challenges were: PBS or 100 μg OVA in 50 μl given daily intratracheally days 15–24. On day 27 airway responsiveness was measured invasively and BAL performed, Panels A and B respectively. Panels C and D, i.p. single injection was: PBS or 50 μg OVA + 4 mg alum on day 0, then airway challenges were: PBS or 50 μg OVA in 50 μl given intranasally days 8, 9 and 10. On day 11 airway responsiveness was measured invasively and BAL performed, Panels C and D respectively. Panels E and F, i.p. injections were: PBS or 20 μg OVA + 4.25 mg alum on days 0 and 14, then airway challenges were: 20 min exposures to aerosolized PBS or 1% OVA on days 28, 29 and 30. On day 31 airway responsiveness was measured invasively and BAL performed, Panels E and F respectively. Each set of panels depicts 2 pooled experiments; total number of mice/group was 6–9.

Suspecting that the importance of the basal IL-4 produced by NKT cells may depend on the dose and type of allergen used to induce disease, we first used models of disease based on the OVA immunization protocols that were used by most publications that described the importance of NKT cells in the induction of AAD. Consequently, we immunized BALB/c WT and CD1d^-^ mice i.p. with 100 μg of OVA plus 4 mg of alum on days 0 and 7, then challenged these mice intratracheally (i.t.) daily with 100 μg of OVA on days 15–24 and then again on day 26. Mice were evaluated for AHR by unrestrained barometric plethysmography on day 25 (S1 Fig) then sacrificed on day 27 with invasive measurement of AHR by forced oscillation and bronchoalveolar lavage (BAL) (Fig 3A and 3B). Because induction of severe OVA-based disease was not impeded by CD1d-deficiency, we wanted to assess a less intense OVA-based model that has been reported to critically depend on NKT cells. Thus, WT and CD1d^-^ mice were injected once i.p. with PBS or 50 μg of OVA plus 4 mg of alum on day 0 and challenged with PBS or 50 μg OVA intranasally on days 8, 9 and 10. On day 11 animals were sacrificed with invasive measurement of AHR by forced oscillation and BAL. Fig 3C and 3D show that AHR and pulmonary eosinophilia were still induced independently of CD1d. Because we suspected that an NKT contribution to AAD might be seen in even milder disease, we compared WT and CD1d^-^ mice in a model in which OVA was delivered by aerosol, rather than through i.t. inoculation. For this model, mice were primed i.p. with PBS or 20 μg of OVA plus 4.25 mg of alum on days 0 and 14, then exposed to aerosolized 1% OVA solution for 20 minutes on days 28, 29 and 30. On day 31 animals were sacrificed with invasive measurement of AHR by forced oscillation and BAL. Fig 3E and 3F show that AHR and pulmonary eosinophilia were again induced independently of CD1d.

### NKT cells do not promote AAD when limiting doses of HDM are administered 6 or 9 times intratracheally

**Fig 4:**
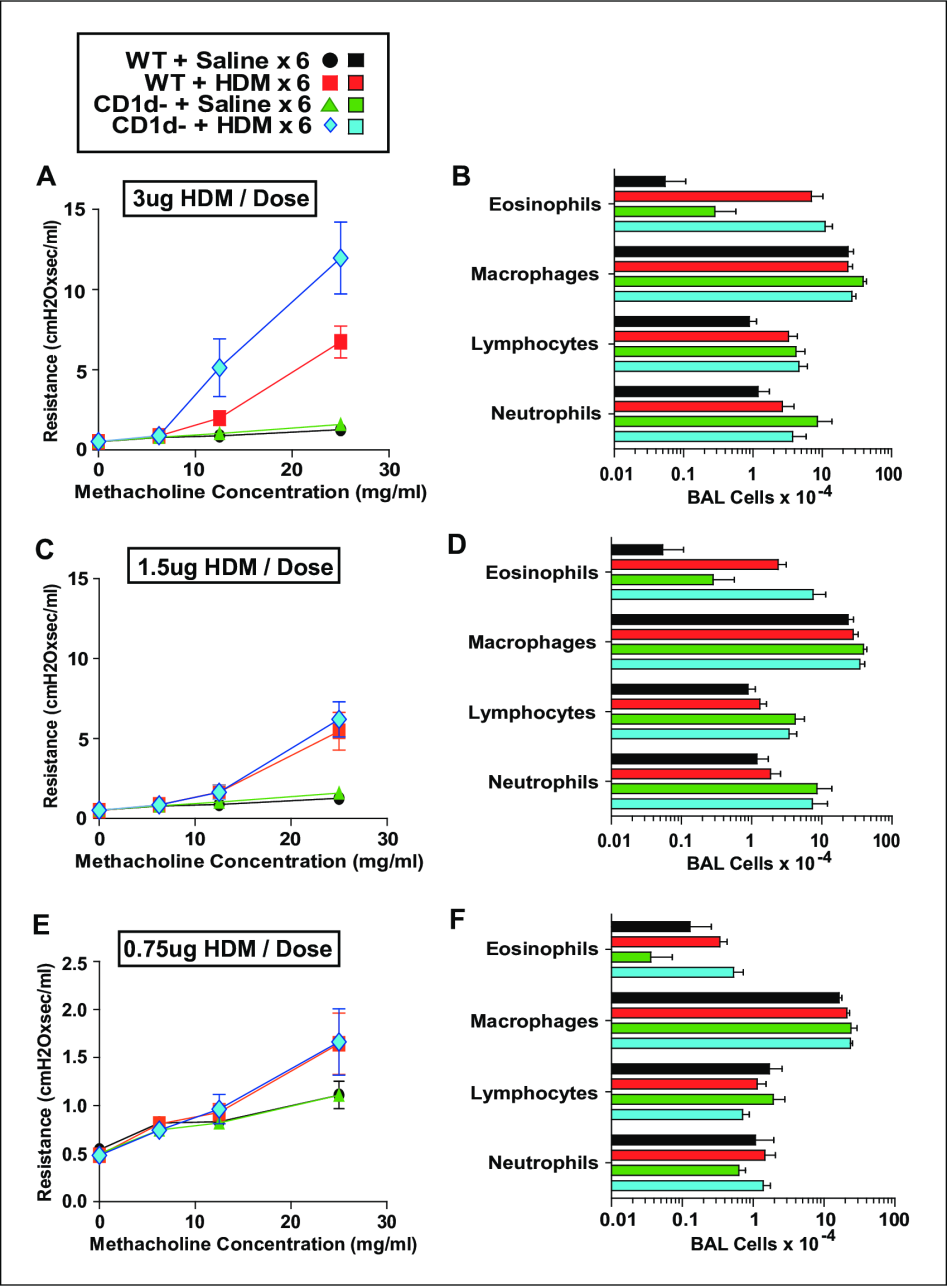
NKT cells do not promote AAD when HDM is administered i.t. at limited doses 6 times. Measurement of AHR (left panels) and cell counts of BALF (right panels) in BALB/c WT and CD1d^-^ mice that were immunized intratracheally with 3 μg (Panels A and B), 1.5 μg (Panels C and D) or 0.75 μg (Panels E and F) HDM every other day 6 times. Data pooled from 2 experiments. Panels A and B, 14 mice/HDM-group; Panels C and D, 10 mice/HDM-group; Panels E and F, 8 mice/HDM-group.

**Fig 5:**
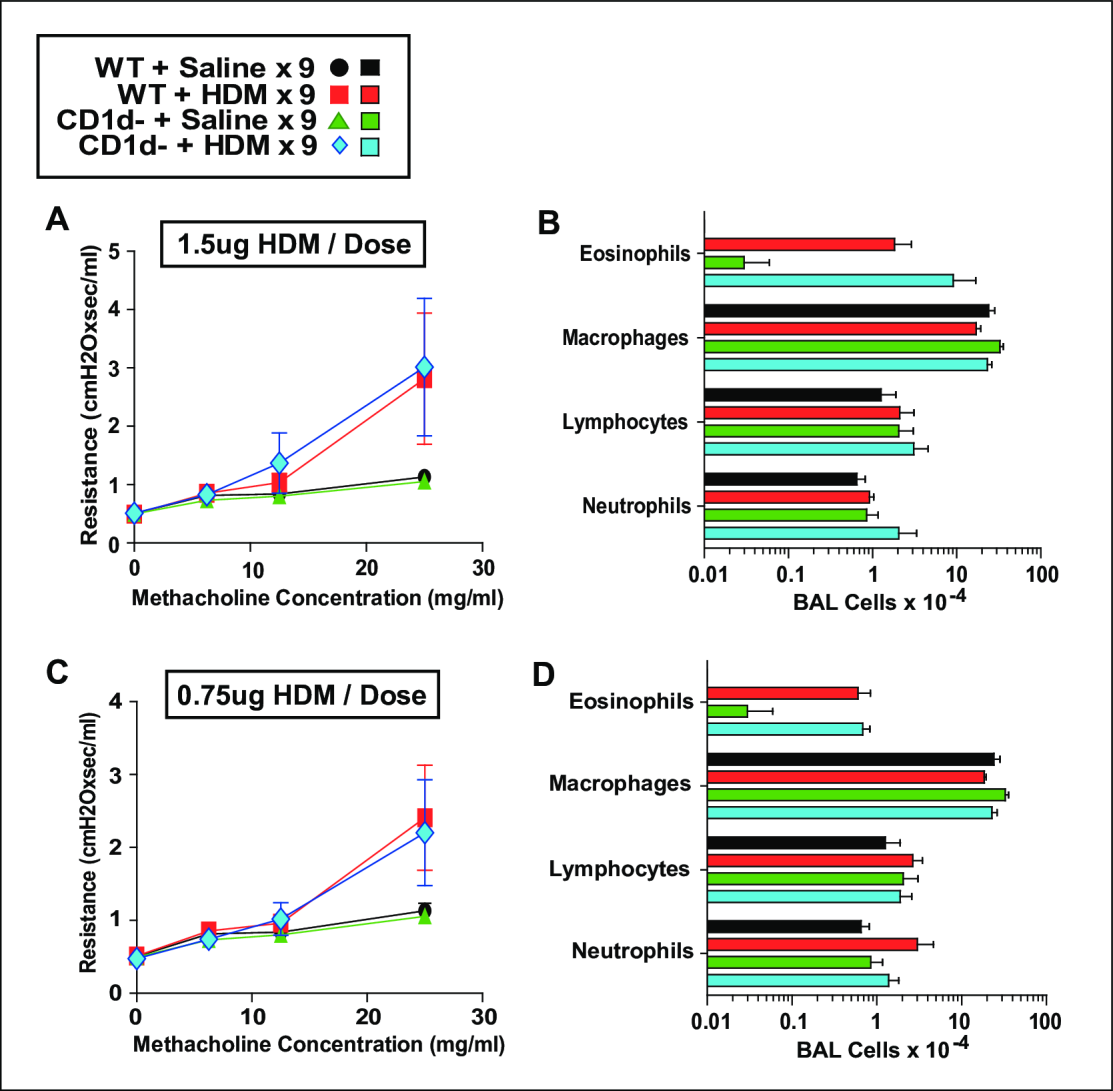
NKT cells do not promote AAD when HDM is administered i.t. at limited doses 9 times. Measurement of AHR (left panels) and pulmonary eosinophilia (right panels) in BALB/c WT and CD1d^-^ mice that were immunized intratracheally with 1.5 μg (Panels A and B) or 0.75 μg of HDM (Panels C and D) every other day 9 times. Data pooled from 2 experiments, 4–6 mice/group.

Because we did not identify an OVA-based model that highlighted the importance of NKT cells in the induction of AAD, we evaluated the NKT contribution to disease induced by HDM, a more clinically relevant allergen that induces disease when it is administered to the airway of naïve mice, obviating the need for antecedent peritoneal sensitization. Inoculating the lower airway with small repetitive doses of this potent allergen allows tight titration of the intensity of sensitization and airway disease. Consequently, we inoculated WT and CD1d^-^ mice i.t. every other day with saline, or 0.75, 1.5, or 3 μg of HDM. Mice were assessed invasively for AHR and pulmonary eosinophilia 1 day after their last inoculation (Fig 4). Minimal, moderate and more severe AAD was successfully induced by the low, higher and highest HDM doses, respectively, but CD1d-deficiency did not decrease either AHR or pulmonary eosinophilia in any treatment group. Given that very minimal disease was induced in the mice that were treated with 0.75 μg of HDM/dose, we wondered if the effect of a decreased basal IL-4 level would become more apparent over time. Thus, we administered saline, 0.75 or 1.5 μg of HDM i.t. every other day to WT and CD1d^-^ mice 9 times rather than just 6. Again, CD1d-deficiency did not impair the development of AAD (Fig 5).

### Anti-CD1d mAb effectively prevents responses to α-GalCer but does not prevent OVA-induced AHR

**Fig 6:**
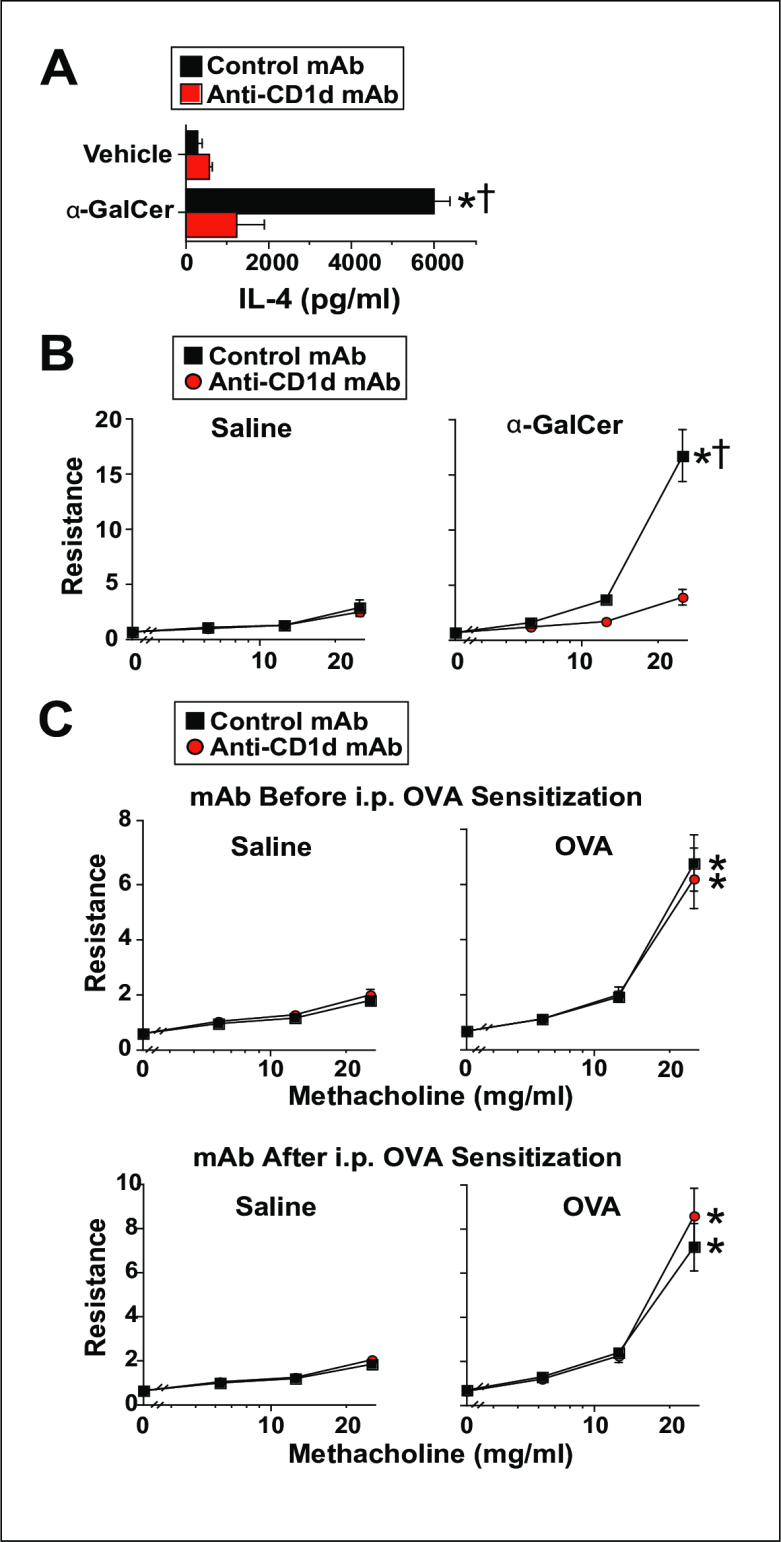
Anti-CD1d mAb effectively prevents responses to α-GalCer but does not prevent OVA-induced AHR. Panel A, BALB/c WT mice were injected i.p. with 500 μg of anti-CD1d or isotype control mAb and treated i.t. with saline or 2 μg of α-GalCer. IL-4 production was measured by IVCCA during the subsequent 24 hrs. 1 experiment with 6 mice/group; a second experiment (not shown) gave compatible data. Panel B, BALB/c WT mice were injected i.p. with 500 μg of anti-CD1d or isotype control mAb, then challenged i.t. with 2 μg of α-GalCer and evaluated invasively 2 days later for AHR. 2 experiments pooled, 12 mice/group. Panel C, BALB/c WT mice were immunized i.p. with OVA/alum, then treated i.t. with saline or 12.5 μg of OVA on days 7, 8 and 9, then interrogated invasively for AHR on day 10. Additionally, the mice were injected i.p. with 500 μg of anti-CD1d or control mAb on days -1, 2, 5 and 9 (Before Sensitization groups) or on days 6 and 9 (After Sensitization groups). 2 experiments pooled 6–11 mice/group. * = p <0.05 as compared to saline-immunized mice. † = p < 0.05 as compared to anti-CD1d mAb-treated mice.

Because our prior experiments were based upon congenital absence of CD1d and NKT cells, we considered that an immunologic compensatory mechanism may have developed in the CD1d^-^ mice that might to explain why AAD was not affected by the absence of NKT cells. Thus, we sought to acutely block antigen presentation to NKT cells by administering an anti-CD1d mAb. To validate the function of this Ab, anti-CD1d mAb or isotype control mAb was injected, followed by i.t. saline or α-GalCer. The IL-4 production in the subsequent 24 hrs was measured by IVCCA, while airway responsiveness was measured in other mice 2 days after their i.t. inoculation. Anti-CD1d treatment provided a striking decrement in both the production of IL-4 and AHR induced by α-GalCer (Fig 6A and 6B). In contrast, anti-CD1d mAb failed to suppress the development of AHR in mice immunized with a low dose of OVA when the mAb was given before antigen-sensitization or just before airway inoculation (Fig 6C).

### HDM increases IL-4 transcription by pulmonary conventional CD4^+^ T cells and not by pulmonary NKT cells

**Fig 7:**
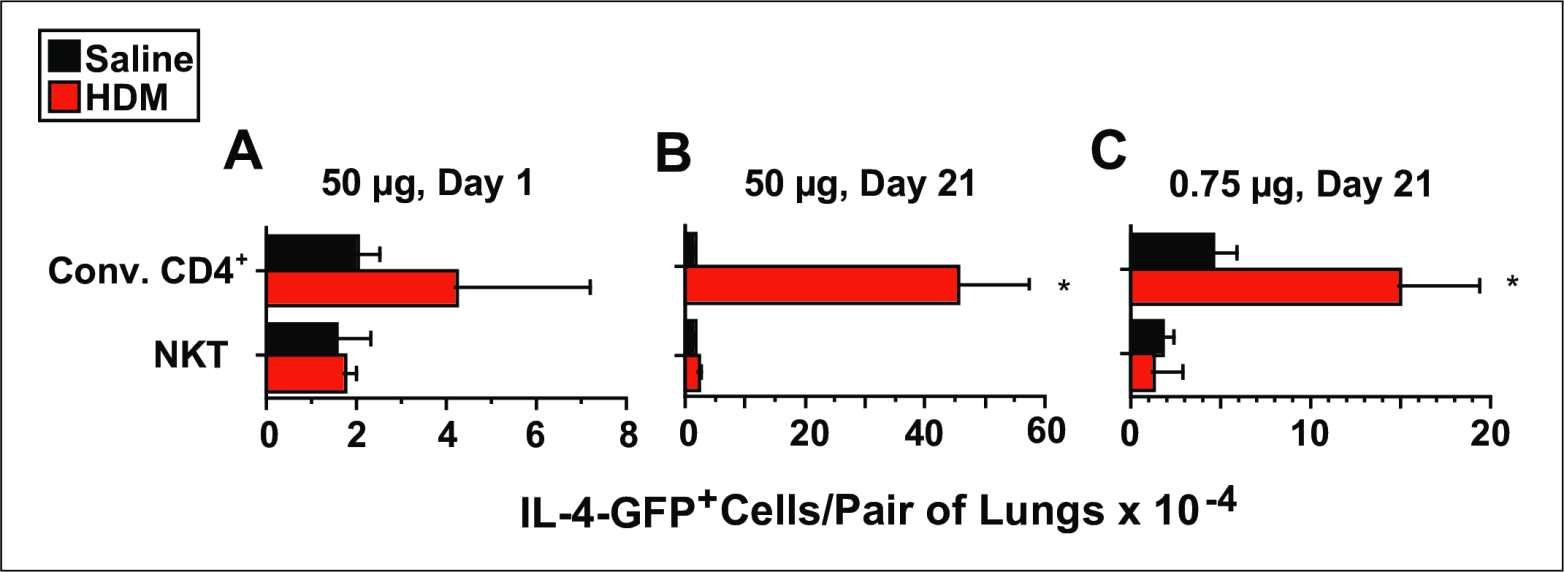
HDM induces IL-4 transcription by pulmonary conventional CD4^+^ T cells and not by NKT cells. 4-get mice (BALB/c background) were inoculated i.t. with: saline or 50 μg HDM once (Panel A), saline or 50 μg HDM 3 times per week for 3 weeks (Panel B), or mice received saline or 0.75 μg of HDM 3 times per week for 3 weeks (Panel C). Lungs were digested 1 day after the last dose of HDM, and flow cytometry was used to determine the total number of NKT cells and conventional CD4^+^ T cells that contained GFP. 3–4 mice/group in 1 experiment for Panels A and C; 6 mice/group from 2 pooled experiments for Panel B. * = p <0.05 as compared to saline-immunized mice.

Because we could not identify a model in which NKT cells contributed to AHR or pulmonary eosinophilia on these macroscopic and microscopic levels, we wondered if the induction of AAD would be associated with signs of cellular activation within NKT cells. To assess this we employed “4-get” mice which synthesize GFP via an internal ribosome entry site when the IL-4 gene is transcribed. Increased GFP was not identified in either conventional CD4^+^ T cells or NKT cells 1 day after a single i.t. inoculation with 50 μg HDM as compared with inoculation with saline (Fig 7A). However, 1 day after the last of 9 i.t. inoculations with 50 μg of HDM, a large increase in the total number of GFP^+^ conventional CD4^+^ T cells was observed in the lungs, without any change in the number of GFP^+^ NKT cells as compared to saline-inoculated mice (Fig 7B). Similar, but less striking, changes were observed 1 day after the last of 9 i.t. inoculations with 0.75 μg of HDM (Fig 7C).

## Discussion

The generation of an allergic response within the airway of mammals has been shown to depend on increased production of type 2 cytokines [20], typically in response to protein antigen. The immunologic cells and molecular mediators that induce such cytokine-production, however, remain an area of intense investigation. Several factors derived from cells considered immunologically innate have been shown to induce a type 2 response in the adaptive immune system [45–52]. Yet interestingly, genetic studies of humans with atopic disease have often reported that increased disease-risk is associated with gain-of-function polymorphisms in genes associated with the adaptive immune system [10, 11].

With this in mind, we took interest in our earlier experiments that showed a distinct decrement in the basal IL-4 levels of CD1d-deficient mice, which lack NKT cells. Because NKT cells are a relatively rare cell type in the lungs of mice with AAD and because IL-4 is a critical humoral factor in the differentiation of Th2 cells from naïve T cells, we postulated that NKT cells might promote the induction of AAD, not by acting as effector cells, but rather by providing a milieu that more favorably supports a type 2 immunologic response. Additionally, we postulated that this mechanism could provide a good explanation for the disparity found in the experimental results of different laboratories that have studied the role of the NKT cell in AAD.

Using several approaches, our experiments show that NKT cells are unequivocally not required for the development of AAD, whether mild or severe, or provoked by a relatively weak antigen or a potent clinically relevant allergen. An implication of these findings is that our BALB/c WT mice are not assisted in the generation of a type 2 response by the substantial amount of IL-4 provided by their NKT cells, or possibly by some other cell type that depends also on the CD1d molecule or the NKT cell population itself. This suggests that the “rate limiting step” for the allergic response may lie in more innate pathways that may precede the initial Th2 differentiation.

Experimentally there are three general ways to study the functional significance of NKT cells *in vivo*: 1) delete gene segments critical for synthesis of their characteristic TCRs, 2) delete the CD1d genes that allow thymic selection and 3) administer antibody that prevents CD1d from binding the TCRs of NKT cells. We chose to utilize the latter two approaches because they provide for a more comprehensive elimination of NKT subsets. For example, while the *Traj18* gene segment is required for invariant NKT development[31, 53], deletion of this gene segment still permits development of more diverse NKT subsets that yet remain dependent on CD1d[54, 55]. Additionally, we did not pursue experiments with mice deficient in TCR gene segments because the groups that have reported a potentiating role for NKT cells in AAD have showed such an effect whether CD1d-deficiency or TCR gene segment deficiency was used to eliminate NKT cells.

The induction of allergic disease in laboratory animals can be influenced by several environmental factors. For example, the presence or absence of *Helicobacter muridarum* [56], segmented filamentous bacterium [57], *Lactobacillus reuteri [57]*, *Fusobacterium varium* [58] and *Bacteroides spp*. [59] affects murine immunity and inflammation, as does the composition of bedding material used in cages [60]. Additionally, differences in intestinal flora can have effects on the mucosal immune system in CD1d-deficient mice that are not observed in WT mice [61].

Likewise, genetics can potently affect an immunologic response, as exemplified by differences between BALB/c and C57BL6 mice in numerous models of disease [62–67]. Indeed, when performing our initial experiments, we found that CD1d-deficient mice showed a mild decrement in AAD compared to wild-type mice only when the very lowest doses of allergen were used to induce AAD. Unwittingly we were comparing strains of mice with ~8% difference in genetic background. After equalizing the genetic backgrounds of the WT and CD1d-deficient mice, we found no difference in AAD between these types of mice.

To provide perspective regarding these conclusions, we extensively reviewed publications that investigated the role of the NKT cell in allergic airway disease and asthma. The essential information and conclusions from each paper that relate to NKT cell participation in allergic airway disease and asthma are shown in S2 Fig for mouse studies [27–37, 68–98] and in S3 Fig 3 for human and non-human primate studies [38–41, 99–108].

Of 42 mouse studies reviewed, 23 used α-gal-cer or another agent known to target NKT cells to influence the development of airway disease. Although the results of these experiments may be relevant to asthma pathogenesis, they do not directly address whether predominantly protein allergens act through NKT cells to induce disease [32, 33, 35, 68, 69, 71–73, 77, 78, 81, 83, 84, 87, 88, 90–96, 98]. Additional studies stimulated mice with purified cytokines and/or other non-protein pro-inflammatory agents, such as ozone [79, 80, 82, 91]. Of the studies, which reported experiments that stimulated mice with purified proteins, such as OVA, or clinically relevant allergens, such as house dust mite extract, 24 (including 7 from 1 group) supported the importance of NKT cells in allergic airway disease induction. 12 studies from 12 different groups gave predominantly negative results (not including our own). And, 6 yielded results that were mixed or equivocal. This may overestimate the ratio of positive to negative data, because of the bias against publishing negative results. Importantly, results did not correlate with mouse strain used (usually BALB/c or C57BL/6); this makes it unlikely that subtle genetic drift could explain the striking differences found in these studies [85, 98]. Consistent with this, our negative results were obtained with CD1d-deficient mice from the same commercial vendor (Jackson) that supplied the mice used in many of the positive studies.

Additionally, both positive and negative results have been obtained with a variety of disease models that differed in: the antigens used, the routes through which mice were sensitized and the method used to reduce or eliminate NKT cells. This suggests that the difference in results of these studies were not caused by differences in the experimental methods used to test the importance of NKT cells in allergic airway disease.

Because experimental results within publications and their parent institutions were generally consistent across different mouse strains immunized with different allergens using different protocols, we favor the view that a variable that differs more between institutions than within an institution may be most likely to explain the differences in NKT cell significance in allergic airway disease. In this regard, it is relevant that differences in the gut microbiome have been shown to influence susceptibility to murine and human bowel disease and to be influenced by CD1d-deficiency [61, 109]. Microbiome differences might have also influenced the results of studies of NKT cells in human asthma, some of which show increased numbers of airway NKT cells in individuals with this disease, while others have failed to find a significant or substantial difference (S3 Fig). In the one study of non-human primates in which an anti-CD1d monoclonal antibody was used to block the effects on NKT cells on the response to airway immunization with a potent antigen, the treatment actually increased pulmonary eosinophilia and had no appreciable effect on airway responsiveness.

Testing our hypothesis that the variability in NKT cell significance in allergic airway disease reported in the literature results primarily from differences in the microbiome would be both difficult and expensive. Probably the best way to study this would for several labs from each camp to verify the genetics of their mice and then determine the composition of their airway and intestinal microbiomes. If consistent correlations are observed between bacterial species and the significance of NKT cells, this hypothesis could be further tested by reconstituting antibiotic-treated or germ-free mice with those species and assessing NKT cell significance. It is not yet clear, however, that even positive results could be applied to the treatment of human asthmatics, because it has not been shown that NKT cell depletion can suppress established disease (even in laboratories that find these cells to be important in the pathogenesis of murine allergic airway disease) or that important specific microbiome differences in mice can predict similar differences in humans.

## Conclusions

The implication of these observations in the study of NKT cell’s relevance to the induction of AAD, is that relatively modest changes in the mice, or the facilities used to house them, may differentially provide significance to the IL-4 produced by NKT cells in the basal state. These considerations are not without clinical significance given the degree of genetic variability and diversity in the flora of human populations across the world. While our results suggest that NKT cell inhibition would likely fail as a treatment for established allergic asthma, such inhibition may be helpful in circumstances when NKT cells secrete large amounts of type 2 cytokines during initial allergen presentation.

## Supporting information

S1 Fig Airway responsiveness as measured by non-invasive unrestrained plethysmography through methacholine challenge in mice treated with high doses of OVA. WT and CD1d- mice were injected i.p. with either PBS or 100 μg OVA + 4 mg alum on days 0 and 7, then treated intratracheally with either PBS or 100 μg OVA in 50 μl on days 15–24. On day 25 airway responsiveness was measured by unrestrained plethysmography. * = p <0.05 as compared to saline-treated mice.
(PDF)

S2 Fig Studies of murine allergic airway disease. (TIF)

S3 Fig Studies of human asthma and primate allergic airway disease. (TIF)
