# Supplementary figures and images for: NKT cells contribute to basal IL-4 production but are not required to induce experimental asthma
Source: PLoS One. 2017 Nov 28;12(11):e0188221. doi: 10.1371/journal.pone.0188221 (PMC5705134; doi:10.1371/journal.pone.0188221)

Supplemental Figure 1.

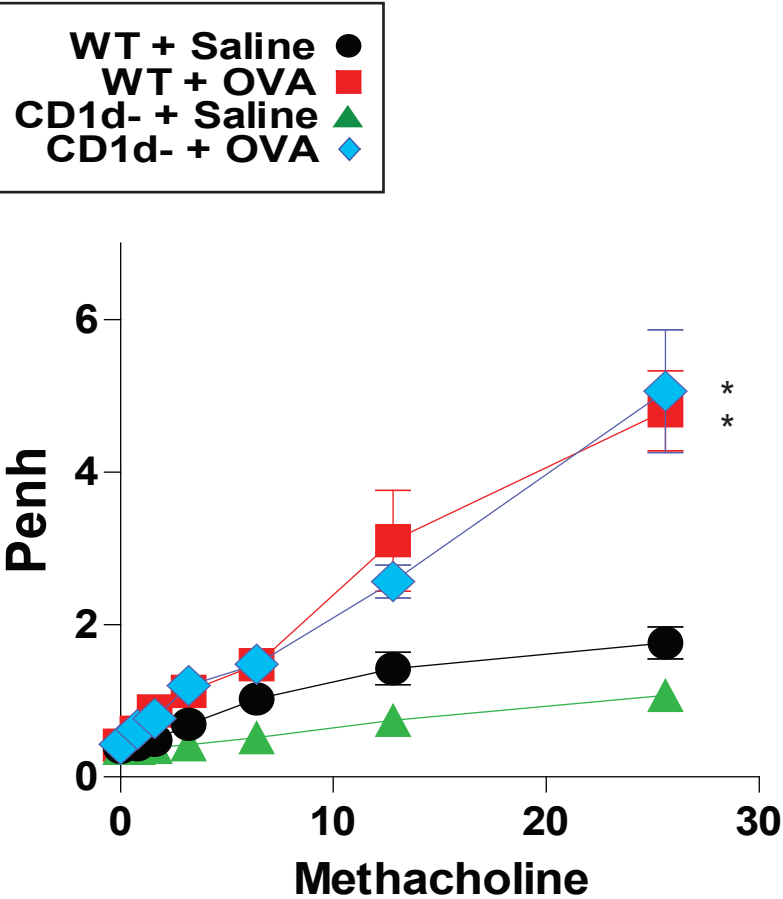

Supplement: S1 Fig — WT and CD1d- mice were injected i.p. with either PBS or 100 μg OVA + 4 mg alum on days 0 and 7, then treated intratracheally with either PBS or 100 μg OVA in 50 μl on days 15–24. On day 25 airway responsiveness was measured by unrestrained plethysmography. * = p <0.05 as compared to saline-treated mice. (PDF) [file pone.0188221.s001.pdf]

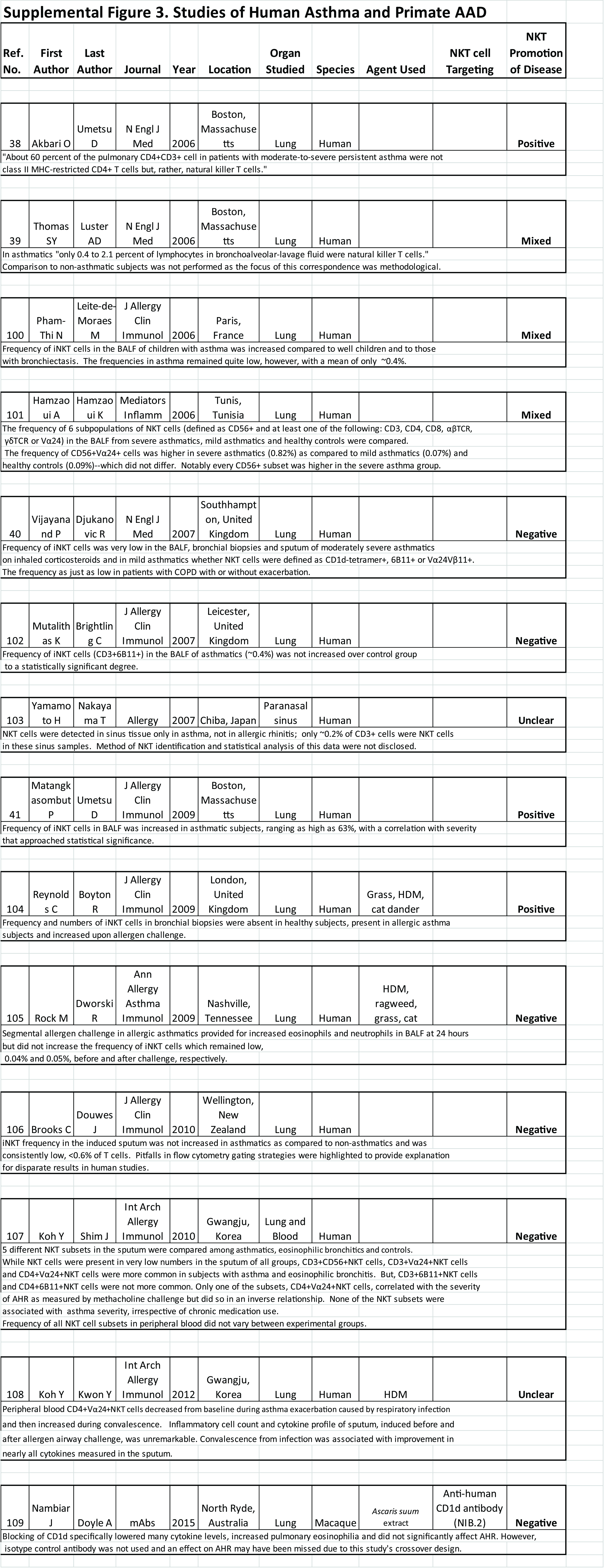

Supplement: S3 Fig — (TIF) [file pone.0188221.s003.tif]
